# Pediatric Colonic Xanthomas, a Previously Unreported Colonoscopic Finding

**DOI:** 10.1097/PG9.0000000000000329

**Published:** 2023-06-09

**Authors:** Jon A. Vanderhoof, Jeffrey D. Goldsmith, Rosemary Pauley, Victor L. Fox

**Affiliations:** From the * Department of Gastroenterology, Harvard Medical School, Boston Children’s Hospital, Boys Town National Research Hospital, Boys Town, NE; † Department of Pathology, Boston Children’s Hospital, Harvard Medical School, Brigham and Women’s Hospital, Boston, MA; ‡ Department of Pediatric Gastroenterology, Boys Town National Research Hospital, Boys Town, NE; § Department of Gastroenterology, GI Endoscopy and Procedure Unit, Division of Gastroenterology, Hepatology, and Nutrition, GI Consultant, Vascular Anomalies Center, Boston Children’s Hospital, Harvard Medical School, Boston, MA.

**Keywords:** colon, colonoscopy, polyps, xanthoma

## Abstract

Gastrointestinal xanthomas are benign, usually sessile, polypoid lesions occasionally incidentally seen in adults, usually in the stomach, but have not been reported in the large intestine in children. We identified xanthomas in the sigmoid colon of the 15-year-old girl confirmed histologically. Our findings suggest that colonic xanthomas may occur as an incidental finding in pediatric patients. They have a characteristic visual and histologic appearance but do not appear to be associated with any symptoms or illness and do not require follow-up.

## INTRODUCTION

**FIGURE 1.**
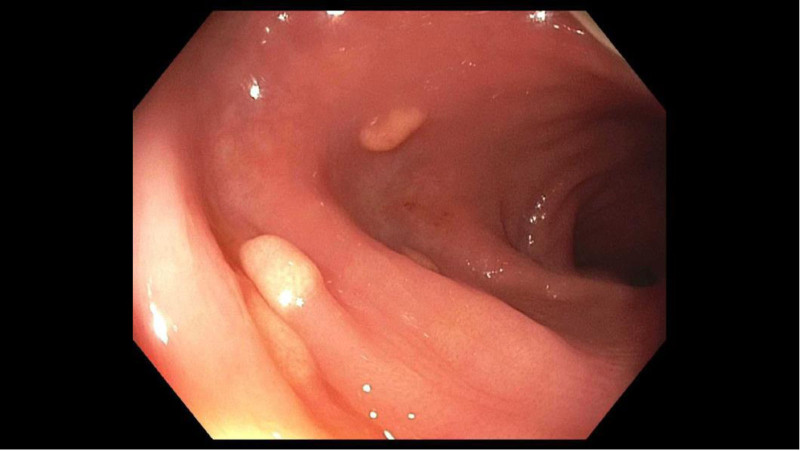
Small, smooth, yellowish polypoid lesions in the sigmoid colon.

Xanthomas are typically cutaneous lesions demonstrating the accumulation of fatty tissue in the dermis and subcutaneous tissues. These generally appear yellow or white in color and are histologically composed of a dermal infiltrate of lipid-laden foamy histiocytes (see Fig. 1). Gastrointestinal xanthomas are seen primarily in older adults and are characterized by abundant lipid-containing histocytes present in the lamina propria (1). They are present most commonly in the stomach (2) and less often in the esophagus, duodenum, and colon. The occurrence of gastrointestinal xanthoma in children is rare but there has been a report of a gastric xanthoma (3,4). We herein report what appears to be the first case of colonic xanthoma in a young female.

## CASE DESCRIPTION

**FIGURE 2.**
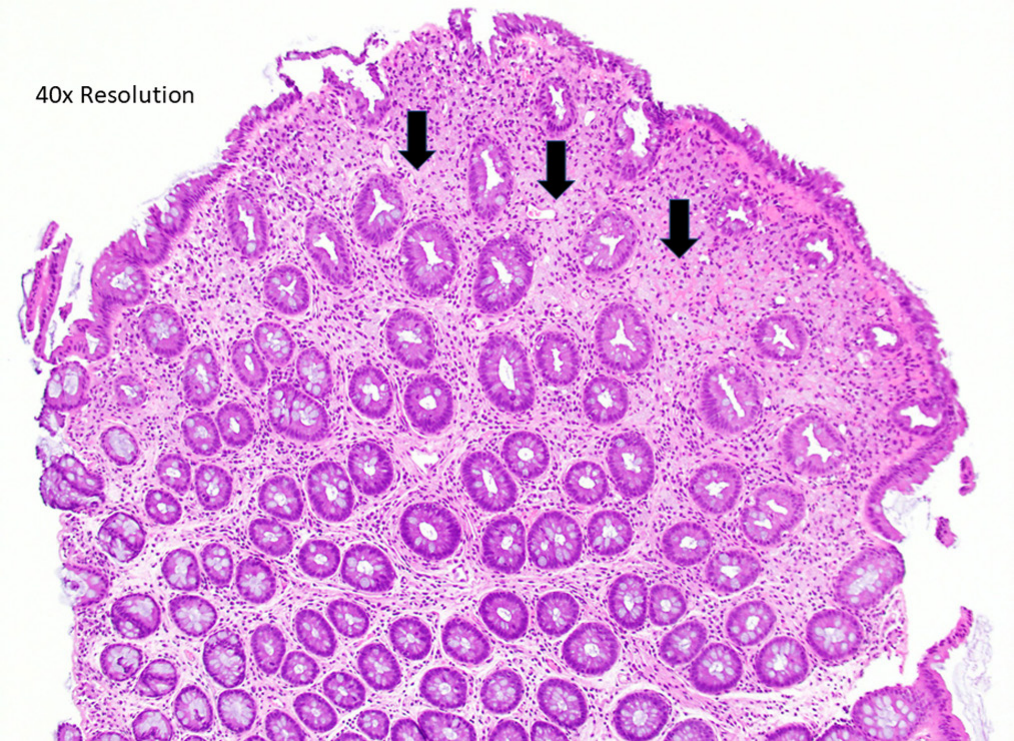
Photomicrographs showing polypoid tissue with abundant lipid-containing histocytes present in the lamina propria characteristic of xanthoma.

**FIGURE 3.**
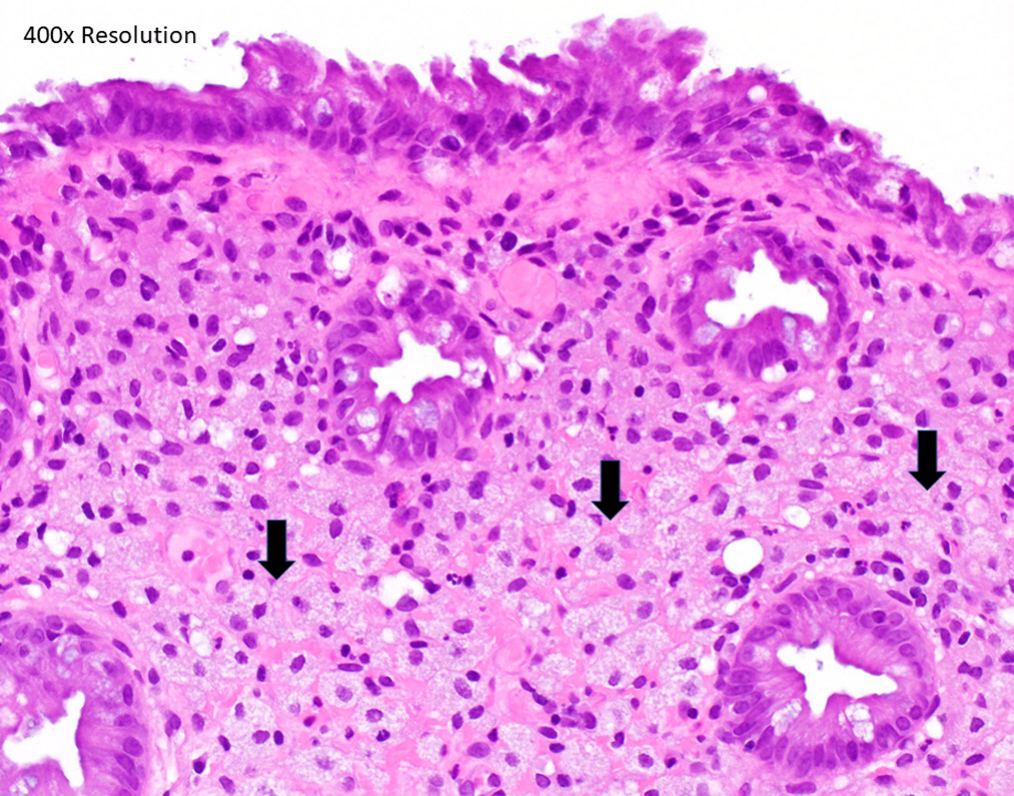
High power photomicrograph of Figure 2.

A 15-year-old female presented to our pediatric gastroenterology clinic with periumbilical, crampy abdominal pain, weight loss, constipation, and bloating. Because of a family history of inflammatory bowel disease, laboratory studies were performed. These demonstrated a hemoglobin of 9.1 milligrams per deciliter, with normal levels of tissue transglutaminase, C-reactive protein, and fecal calprotectin. An upper gastrointestinal endoscopy at that time demonstrated the presence of *Helicobacter pylori* gastritis, which required 2 rounds of treatment for the resolution of symptoms and anemia. The patient was subsequently seen 2 years later with a return of abdominal pain and bloating. Because of the family history of inflammatory bowel disease, recurrent anemia, and persistent symptoms, a repeat gastrointestinal endoscopy was performed. The upper endoscopy was normal. Colonoscopy revealed three 1–2 mm smooth yellowish polypoid lesions without any stigmata of bleeding, malignancy, or surrounding gross mucosal abnormalities in the sigmoid colon, while the remaining colon and terminal ileum appeared normal. The lesions appeared inconsistent with any of the common colonic polypoid lesions normally seen in children (see Figs. 2 and 3). Histology showed abundant lipid-laden histiocytes in the lamina propria consistent with xanthomas. Biopsies of the nonpolypoid mucosa were histologically unremarkable.

## DISCUSSION

Gastrointestinal xanthomas in adults are most frequently seen in the stomach and rarely in the esophagus and colon. The mechanism of the development of xanthomas is not known. In the colon, it has been speculated that they may be related to chronic constipation and possibly indicative of prior injury (1). In this sense, these lesions are probably different from those in the upper gastrointestinal tract. The cause of the xanthomas in our patient is unknown. There was a history of constipation and bloating, although there was no evidence of hemorrhoids, trauma, or prolapse. Our patient’s constipation was thought to be insignificant. Straining upon defecation, as often seen with stercoral ulcer formation, was not reported. However, the sigmoid location of the polyps would be consistent with a history of constipation. As multiple random biopsies were obtained from the remainder of the colon and there were no other findings of inflammation, we concluded that these lesions were not likely to be part of an inflammatory process. Likewise, the patient did not have hyperlipidemia, and there was no family history of hyperlipidemia or heart disease.

This report confirms that colonic xanthomas may occur in children as well as adults, their appearance is somewhat characteristic, and they are likely to be benign and inconsequential. The location of the lesions in our case was inconsistent with the most reported locations in most adults (5). As this is the first reported pediatric case, the incidence appears to be quite low in children, but it is possible that a number of these lesions have gone undetected or were simply not reported. At present, we lack enough data to provide recommendations for follow-up; however, the benign nature of the lesion in adults suggests further evaluation is not indicated. Our report suggests that xanthomatous polyps should be included in the differential diagnosis of colonic polypoid lesions in children.

## ACKNOWLEDGMENT

Informed consent was obtained from the mother before publication.
